# Retina of the fruit fly eyes: a detailed simulation model

**DOI:** 10.1186/1471-2202-16-S1-P301

**Published:** 2015-12-18

**Authors:** Aurel A Lazar, Konstantinos Psychas, Nikul H Ukani, Yiyin Zhou

**Affiliations:** 1 Department of Electrical Engineering, Columbia University, New York, NY 10027, USA

## 

The compound eyes of the fruit flies consist of the retina and 4 neuropils. The retina comprises about 700~800 ommatidia, each of which houses 8 photoreceptors, where the phototransduction takes place. Although seemingly simple, each photoreceptor can perform complex computation on their own [1]. Nevertheless, it is not yet clear how individual photoreceptors contribute to an overall spatiotemporal processing of visual scenes. Towards this goal, we constructed a full-scale simulation of the retina on a cluster of GPUs.

**Figure 1:**
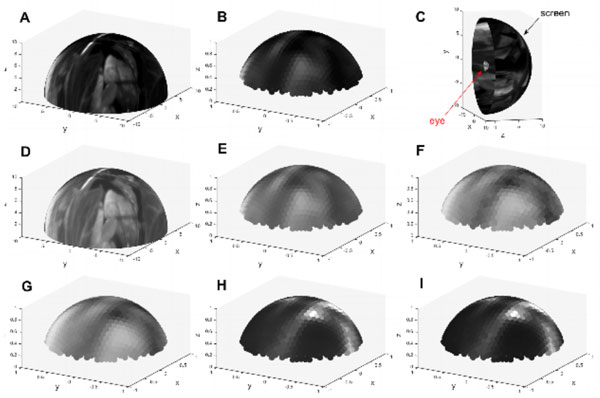
**A. Stimulus presented on a hemisphere screen**. B. Inputs (Number of Photons) to R1 photoreceptors in all ommatidia. C. Geometric relation between eye and screen. D. Gamma corrected screen intensity. E. Gamma corrected inputs to R1 photoreceptors. F. Voltage response of R1 photoreceptors. G. Averaged voltage response of photoreceptor terminals in all cartridges. H. Response of L1 neurons in all cartridges. I. Response of L2 neurons in all cartridges.

The geometry of the eye was taken into account by building a hexagonal array of 721 ommatidia on a hemisphere and assigning a proper optic axis and an acceptance angle to each of the photoreceptors. The visual stimulus was presented on either a hemisphere or a cylinder surrounding the eye, as in many experimental settings (see Figure 1).

The phototransduction process is based on a biochemical model of 30,000 microvilli followed by a conductance based biophysical model of the cell membrane [1]. Here we only consider monochromatic photoreceptors R1-R6 in each ommatidium. Each photoreceptor is described by ~ 450, 000 equations; the total number of equations simulated for the entire retina amounted to about 1.95 billion. All the simulations were performed on 4 Tesla K20X GPUs; it took approx. 7 minutes to finish 1 second of simulation.

After constructing the detailed simulation model, we tested the processing of visual stimuli by the retina circuit. Furthermore, to test the effect of feedback originating in the lamina on the photoreceptors, we linked the retina model with a lamina model [2] in the Neurokernel environment [3]. An example of response of the circuit to natural stimuli is shown in Figure 1. The simulations reveal that composition rules in the lamina circuit affect the spatiotemporal processing carried out by the photoreceptors under the model parameters considered here.

Finally, we scaled up the retina model of the fruit fly to the size of the blow fly to investigate the effect of visual acuity and noise.
